# Laccases in Food Industry: Bioprocessing, Potential Industrial and Biotechnological Applications

**DOI:** 10.3389/fbioe.2020.00222

**Published:** 2020-03-24

**Authors:** Karla Mayolo-Deloisa, Mirna González-González, Marco Rito-Palomares

**Affiliations:** ^1^Tecnologico de Monterrey, Escuela de Ingeniería y Ciencias, Monterrey, Mexico; ^2^Tecnologico de Monterrey, Escuela de Medicina y Ciencias de la Salud, Monterrey, Mexico

**Keywords:** agro-food wastes, by-products, bioprocessing, food industry, laccase

## Abstract

Laccase is a multi-copper oxidase that catalyzes the oxidation of one electron of a wide range of phenolic compounds. The enzyme is considered eco-friendly because it requires molecular oxygen as co-substrate for the catalysis and it yields water as the sole by-product. Laccase is commonly produced by fungi but also by some bacteria, insects and plants. Due it is capable of using a wide variety of phenolic and non-phenolic substrates, laccase has potential applications in the food, pharmaceutical and environmental industries; in addition, it has been used since many years in the bleaching of paper pulp. Fungal laccases are mainly extracellular enzyme that can be recovered from the residual compost of industrial production of edible mushrooms as *Agaricus bisporus* and *Pleurotus ostreatus.* It has also been isolated from microorganisms present in wastewater. The great potential of laccase lies in its ability to oxidize lignin, one component of lignocellulosic materials, this feature can be widely exploited on the pretreatment for agro-food wastes valorization. Laccase is one of the enzymes that fits very well in the circular economy concept, this concept has more benefits over linear economy; based on “reduce-reuse-recycle” theory. Currently, biorefinery processes are booming due to the need to generate clean biofuels that do not come from oil. In that sense, laccase is capable of degrading lignocellulosic materials that serve as raw material in these processes, so the enzyme’s potential is evident. This review will critically describe the production sources of laccase as by-product from food industry, bioprocessing of food industry by-products using laccase, and its application in food industry.

## Introduction

Laccase (benzenediol:oxygen oxidoreductase; EC 1.10.3.2) is a blue-copper oxidoreductase that catalyze the oxidation of wide range of substrates including phenolic compounds with the concomitant reduction of molecular oxygen to water (Nunes and Kunamneni, 2018). Fungal laccases contain two disulfide bonds and four copper atoms distributed in three cupper centers: T1, T2, and T3. Type I (T1) is mononuclear and has an absorption band at around 610 nm, which is responsible for the characteristic blue color of such enzyme. T2/T3 are a trinuclear cluster. The oxidation of the substrate is carried out in T1, through a His-Cys-His tripeptide sequence, the extracted electrons are transferred to the T2/T3 site where the reduction of molecular oxygen to water finally occurs (Osma et al., 2010; Hautphenne et al., 2016; Nunes and Kunamneni, 2018). That is the reason why laccase is considered as a “green tool,” due to it is able to perform the catalysis process using molecular oxygen as the only co-substrate rather than hydrogen peroxide like other oxidoreductases (*v.gr*. lignin peroxidase and manganese peroxidase) (Agrawal et al., 2018). Laccase can cooperate with small compounds called “mediators” and oxidize non-phenolic compounds, so that its activity is not limited only to phenolic compounds (Chio et al., 2019). Mediator is oxidized giving one or more electrons to laccase, after the mediator oxidized form diffuses away from the catalytic pocket, where it is capable of oxidizing the substrate. Mediator is able of oxidizing substrates inaccessible for the laccase due to its small size (Rochefort et al., 2004; Munk et al., 2018). Most biotechnologically useful laccases are fungal origin, however, the enzyme has been found in plants, insects and some bacteria (Nunes and Kunamneni, 2018). Around 150 laccases have been fully characterized. The most studied have been isolated from fungi capable of destroying wood, especially white-rot fungi as: *Pleurotus pulmonarius*, *Pleurotus ostreatus*, *Agaricus bisporus*, *Trametes versicolor*, etc. (Bertrand et al., 2016; Nunes and Kunamneni, 2018). The production of the enzyme can be influenced for the type of culture (submerged or solid state), type of microelements and the source of nitrogen. Generally, the enzyme is produced during the fungi secondary metabolism (Brijwani et al., 2010). Laccases are secreted, with typical molecular weight around of 60 kDa. Typical fungal laccases have an isoelectric point around 4.0, however there are some laccases with basic isoelectric points (Mukhopadhyay and Banerjee, 2015). The optimal temperature of fungal laccases may vary in a range of 40–70°C (Debaste et al., 2018). They are generally glycosylated, which contributes to the high stability of the enzyme (Osma et al., 2010). Owing its versatility, stability, and wide range of substrates; a large number of laccase industrial applications have been explored in the past years, including delignification and brightening of pulp paper, decolorization of dyes in textile industry, bioremediation of soils, organic synthesis of medications, biosensor technology, and in food processing (Upadhyay et al., 2016).

In nature, laccase is secreted by the fungus to access carbohydrates (cellulose and hemicellulose) in the wood through the degradation of lignin (Osma et al., 2010). Lignin, which is a phenylpropanoid biopolymer, it is considered the most abundant polymer in nature (Chio et al., 2019). Lignin has an extremely complex structure; it is recalcitrant and difficult to degrade. In its natural state it has practically no applications, however, it can be burned to get energy. That is why one of the great challenges to allow the use of lignin is its depolymerization. Currently, there are different methods of lignin depolymerization: thermo-chemical, mechanical, chemical, and biological (Chio et al., 2019). Naturally, biological treatments have been classified as “environmental friendly catalyst” since they are developed by microorganisms or enzymes that are generally not toxic (Chio et al., 2019). In addition, biological treatments have low energy requirements, reduced waste streams and low downstream cost (Albornoz et al., 2018). In this way, the role of laccases in the production of bioethanol through the pretreatment of ligninolytic residues has been studied. Since the treatment with laccase allows the degradation of lignin which increases the fermentability of such ligninolytic residues (Agrawal et al., 2018). It is known that with help of mediators, laccase degrades almost 80–90% of lignin structure (Chio et al., 2019). The conversion of lignin moieties into biofuels is one of the objectives of biorefinery (Ponnusamy et al., 2019). In general, biorefinery has been defined as a facility for converting lignocellulosic materials into bioenergy and chemicals with potential industrial significance (Roth and Spiess, 2015; Sperandio and Ferreira Filho, 2019). As mentioned earlier, lignocellulosic materials are very abundant and therefore represent a much more suitable resource as a raw material for biorefineries. Thus eliminating the problem of using feedstock for producing biofuel that can be used in food production (Roth and Spiess, 2015).

In this review paper; we analyzed the production sources of laccase, together with the state of the art in the recovery of laccase from by-products generated mainly by the food industry. The bioprocessing of the food industry by-products using laccase and the potential applications of the enzyme in the food industry are presented. Potential trends and current challenges of the potential application of laccase are critically described.

## Laccase as By-Product From Food Industry

The biological pretreatment of lignocellulosic biomass is cost-effective and eco-friendly. Lignin can be degraded by enzymes secreted through the metabolism of wide variety of fungi. For example, white-rot fungi show more significant effects in the degradation of lignocellulose materials (Ponnusamy et al., 2019). Traditionally, laccase has been produced by white-rot fungi using submerged fermentation batch culture. This strategy is very convenient due it makes possible to control the conditions and enhance the production of the enzyme. Nevertheless, sometimes it can be an expensive process, especially if the enzyme requires a high fold purification. The production of laccase at laboratory level has been widely reported, however in the market, there are only some presentations from fungi available such as *Agaricus bisporus*, *Coriolus versicolor*, *Pleurotus ostreatus*, *Trametes versicolor*, and *Rhus vernicifera* (Osma et al., 2010). A great alternative to obtain laccase from agro-food wastes is the residual compost used for the commercial production of edible mushrooms; because laccase is secreted to the compost during the growth of the fungus.

Royse et al. (2017) reported that in the last 40 years the cultivation of edible mushrooms in the world increased about 30-fold, which gives an idea of the commercial value of this type of food. In 2013, the mushroom production industry was valued about of $63 billion and in *per capita* consumption it exceeds 4.7 kg annually. Cultivated mushroom are saprophytes, capable of growing on lignocellulosic materials as they have the ability to degrade them, such materials are generally abundant and may come from industrial or agricultural wastes (Kertesz and Thai, 2018). Residual compost is available in huge amounts; when 1 kg of mushrooms is produced, about 5 kg of residual compost are obtained (Grimm and Wösten, 2018). Among the main edible mushrooms produced around the world are the genera: *Lentinula*, *Pleutorus*, *Auricularia*, *Agaricus*, and *Flammulina*. *Lentinula edodes* (Wong et al., 2013), *Agaricus bisporus*, and *Pleurotus ostreatus* have been recognized as laccase-producing basidiomycetes and they are produced at industrial scale (Yang et al., 2017).

**FIGURE 1:**
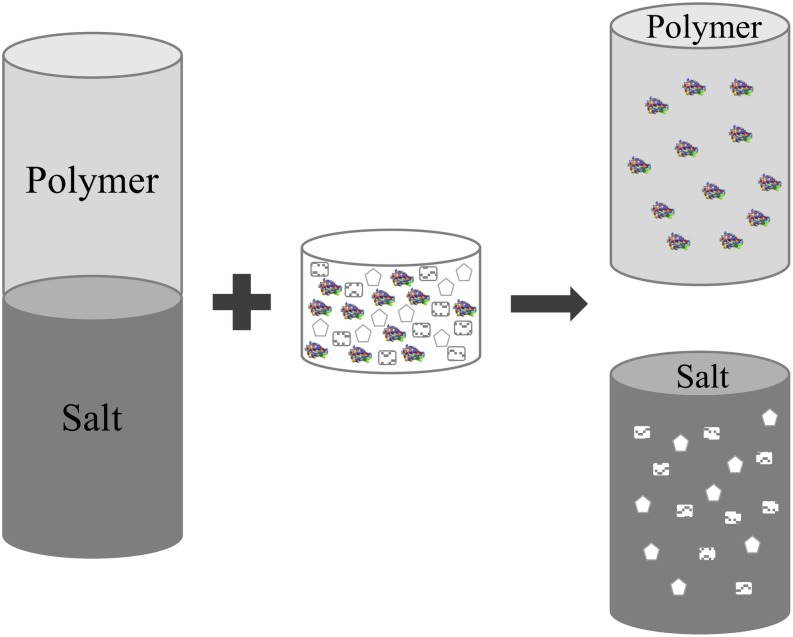
Aqueous two-phase systems (ATPS). ATPS can be formed through the combination of two polymers or a polymer and a salt at critical concentrations. Many parameters are involved in the partition, however in general the enzyme is concentrated in one of the phases.

*Agaricus bisporus* is one of the most produced mushrooms worldwide, capable of using different substrates for its growth (Trejo-Hernandez et al., 2001). The compost used is a complex mixture prepared using horse manure and/or straw added with nitrogen sources such as poultry manure, urea, ammonium nitrate and plaster (Kertesz and Thai, 2018). The production of laccase during the growth of *A. bisporus* on this type of compost has been reported previously so it is known that large quantities of laccase are secreted. Once the fungus production is finished, the compost can be discarded (Trejo-Hernandez et al., 2001; Mayolo-Deloisa et al., 2009). There are some studies demonstrating the presence of laccase in the residual compost. Trejo-Hernandez et al. (2001) evaluated the oxidative capacity of an aqueous crude extract from compost using various phenolic compounds which are laccase typical substrates. On such studies, they worked with an aqueous extract of the residual compost; that is to say, the enzyme was not pure. That represents an advantage, if the future application does not require a high pure laccase, as it is the case of decolorization of textile dyes. Further, the use of aqueous two phase systems (ATPS) for the primary recovery of laccase from residual compost of *A. bisporus* has been reported (see Figure 1; Mayolo-Deloisa et al., 2009). The most commonly used ATPS are formed by two polymers or one polymer and one salt, which mixed in critical concentrations demonstrate incompatibility (Mayolo-Deloisa et al., 2017). The components of each phase are immiscible despite containing about 80% water (molal base). Each phase has different characteristics, which allows them to partition a sample with different molecules (Zaslavsky et al., 2016). They are considered as a primary recovery stage for the separation of biological molecules, due to the large amount of water they contain. ATPS have been used for the treatment of complex samples such as the aqueous extract of residual compost. In that work, polyethylene glycol (PEG) - phosphate ATPS were utilized and the presence of laccase in the PEG rich-phase (top-phase) was proved. It is known that PEG prevents aggregation and gives some stability to proteins, so it was not separated from laccase and the top-phase was used to oxide several Polycyclic Aromatic Hydrocarbons (PAHs), which are generally present in the most recalcitrant fraction of crude oil. The best results were reached with the oxidation of benzo[a]pyrene (BaP), which is formed by a very complex structure difficult to degrade. During the treatment, no interference was observed due to the presence of PEG, on the contrary, a higher oxidation percentage was achieved than the one obtained with the crude extract (Mayolo-Deloisa et al., 2011).

On the other hand, *Pleurotus ostreatus* is one of the most-produced species in the world. It is cultivated mainly on sawdust but it is capable of a rapid conversion of other substrates, including bagasse and cornstalks. Generally the fungus grows by competing with other microorganisms since it is not very common to sterilize the compost before being inoculated (Kertesz and Thai, 2018). The residual compost from *Pleurotus ostreatus* has been used in the production of bioethanol as it is a rich source of sugars such as glucose and xylose (Grimm and Wösten, 2018). Additionally, the presence of laccase in this waste has also been proved. Bertrand et al. (2016) evaluated the potential of different ATPS in the partial recovery of laccase including PEG-phosphate, UCON-salts and polymer-polymer ATPS. The UCON-salts ATPS, specially UCON-(NH_4_)_2_SO_4_, reached a recovery percentage and purification factor up to 98.31% and 9.97, respectively. This demonstrates the potential of systems to recover laccase. UCON is a random copolymer (composed by 50% ethylene oxide and 50% propylene oxide) with a cloud point temperature as low as 40°C. It is a thermoresponsive polymer so it is easier to recover it after a temperature change, where laccase can be separated and the polymer can be recycled (Pereira et al., 2003). Additionally, polymer-polymer systems as PEG10,000/Dextran 10,000 and PEG10,000/Dextran 100,000 have also proved their potential in the recovery of the enzyme; reaching high purification factors. In general, polymer-polymer systems are more expensive, so their application will depend on the final destination of the enzyme.

Laccase can be recovered from residual compost, a lignocellulosic material waste from food industry. After the recovery, laccase can be used for different applications including the degradation of other lignocellulosic materials. It is clear that ATPS represents a viable alternative to be applied in the primary recovery of this enzyme from waste. ATPS-based processes can be formed through a large combination of polymers and salts, are often inexpensive and easy to scale up for commercial purposes.

## Production of Laccase Using Agroresidues

**FIGURE 2:**
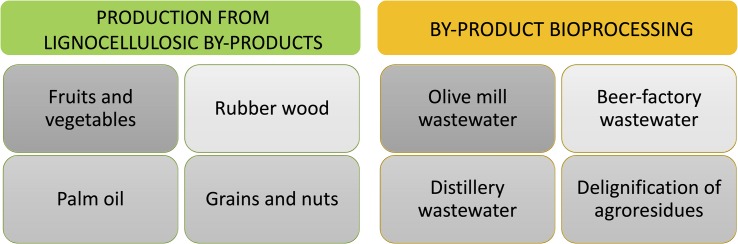
Laccase in food industry. Laccase can be used for the bioprocessing of different food industry by-products, as well as produced in agroresidues by laccase producing fungi.

The generation of waste from the processing of fruits and vegetables represents up to 60% of its production, which highlights the importance of developing new alternatives for the use of this material (Pleissner et al., 2016). There are also other sources of waste production such as those generated by the processing of palm oil and rubber wood that are discharged in large quantities into the environment (Vikineswary et al., 2006). Many of them are lignocellulosic residues. The potential of using such residues in the growth of the *Pycnoporus sanguineus* fungus through solid state fermentation (SSF) has been previously reported, proving that it is a route for the production of laccase (Figure 2). Laccase can be produced with high yields through the degradation of sago and oil palm parenchyma tissue (Rajagopalu et al., 2016). There are also other residues that have been reported for the growth of the *Ganoderma lucidum* fungus, including sunflower seed hulls and rice residues (straw and husk). *G. lucidum* is a laccase-producing fungus, recognized as a medicinal mushroom and highly valued in the market. Pilot-scale studies demonstrated the bioconversion of these substrates during the production process of *G. lucidum*, additionally the presence of laccase in the different crude extracts was also detected. This suggests that there is an area of opportunity in the optimization of enzyme production (Postemsky et al., 2017). There is also evidence of the use of paddy straw and coir pith in combination with biogas digester residue (anaerobically digested plant material) in the cultivation of *Pleurotus ostre*atus and *Pleurotus florida*; reaching high levels of fungus production and demonstrating effectiveness in the degradation of lignin, cellulose, and hemicellulose. Although in this case, the production of laccase was not directly measured, its activity is implicit due to the degradation of lignin (Chanakya et al., 2015). In addition, there are other substrates of agro-industrial origin that can be used for the growth of ligninolytic fungi and therefore for the production of laccase. These may include: barley bran, chestnut shell waste, potato pulp, banana skins, mandarin peels, kiwifruit wastes, and grape seeds (Strong and Claus, 2011).

## Bioprocessing of Food Industry By-Products Using Laccase

Many by-products generated by food industry have high content of phenolic compounds, low pH, as well as high biochemical oxygen demand (BOD) and chemical oxygen demand (COD). Due such complex characteristics is difficult to apply directly a treatment using microorganisms hence enzymatic treatments are more appropriate. Thus, this section will review the main applications of laccase in the processing of by-products from food industry (summarized in Figure 2).

### Olive-Oil Mill Wastewater

The olive-oil production process contributes significantly to the pollution of the Mediterranean area, because it generates a large amount of both solid and liquid wastes with a concentration of organic compounds that is toxic to nature (Osma et al., 2010; Jurado et al., 2011). The biggest problem in olive-oil wastewater treatment is principally related to the low pH, high BOD, COD, the high concentration of phenols (from 1.5 to 8.0 g/L) and other organic substances (Chiacchierini et al., 2004). Chemical treatments have so far been the most used to treat this waste. However, due to the toxicity of the residue, biological treatments directly with ligninolytic fungi and/or their enzymes have been studied (Berrio et al., 2007). Laccase from white-rot fungi as *Pleurotus ostreatus*, *Trametes versicolor, Lentinula edodes, Cerrena unicolor*, and *Pycnoporus coccineus* have been able to oxidize different phenolic compounds present in olive-oil mill wastewaters (Osma et al., 2010), and such activity can be enhanced by enzyme immobilization (Berrio et al., 2007). A strong impact of the enzyme on color removal and phytotoxicity has also been reported (Strong and Claus, 2011).

### Distillery Wastewater

During the fermentation of molasses from sugarcane, ethanol is produced, which leads to the generation of distillery wastewater (Osma et al., 2010). Due to its extreme physicochemical characteristics such as high content of organic and inorganic matter, high values of BOD and COD, acidic pH, high concentration of ash, high temperature, and dark color; distillery wastewater is one of the most polluting waste and difficult to degrade (Kharayat, 2012). The effectiveness of the laccase in removing phenolic compounds and the color of different distillery wastewater has been evaluated, however it has been found that the fungal culture is more effective than the laccase alone (Strong and Burgess, 2008). Which suggests that this residue can also be used for the production of ligninolytic enzymes (as laccase) using it as a substrate for the growth of white-rot fungi.

### Beer-Factory Wastewater

Another residue contaminated with a high load of polyphenolic compounds (especially tannins) is that generated from beer production (Chiacchierini et al., 2004). Tannins are one of the very abundant phenolic compounds in plants, only surpassed by lignin. There are studies that shown the efficiency of *Corolopsis gallica* in the decolorization and a reduction of the COD of a beer-industry effluent containing a high tannins proportion (Yagüe et al., 2000). *C. gallica* fungus is able to grow on beer-factory wastewater because it has a high tolerance to tannins which even allows the production of laccase. It has been observed that when this residue is added to the fungus culture, laccase secretion is increased (Chiacchierini et al., 2004).

### Delignification of Agroresidues

Laccase is one of the enzymes that fits very well in the circular economy concept, this concept has more benefits over linear economy; based on “reduce-reuse-recycle” theory (Agrawal et al., 2018; Giacobbe et al., 2018). In that sense and as mentioned before, laccase can be recovered from waste materials after industrial mushroom cultivation but can also be used to treat lignocellulosic residues generated by agro-industrial activities (Jurado et al., 2011). Agroresidues contain high concentrations of cellulose and hemicellulose that can be a raw material used in biorefinery processes (Giacobbe et al., 2018). It has been reported that the use of laccase for the degradation of lignin present in food residues, such as apple pomace and coffee silverskin, allows the hydrolysis of hemicellulose generating a high sugar content (Giacobbe et al., 2018). In the same way the use of laccase from *Myrothecium verrucaria* in the delignification of agroresidues (like wheat straw, rice straw, and sugar cane bagasse) has been reported, reaching efficient delignification in the absence of mediators (Agrawal et al., 2018).

## Food Industry Application of Laccase

Over the years, laccase has been used in the food industry especially in the determination of different phenolic compounds that can affect the quality of some products. Table 1 summarizes some of the most important applications of laccase in the processing of foods.

**TABLE 1:** Direct applications of laccase in food processing industry.

**Food application**	**Objective**	**References**
Beverage processing	Enhance or modify color appearance	Osma et al., 2010
Wine stabilization	Selective polyphenol removal	Minussi et al., 2007
Beer stabilization	Removal of oxygen in finished beer	Osma et al., 2010
Baking	Increase strength, stability, and reduce stickiness improving machinability of the dough	Selinheimo et al., 2006; Osma et al., 2010
Sugar beet pectin gelation	Crosslink the beet pectin through the oxidative coupling of the feruloyl groups	Micard and Thibault, 1999; Minussi et al., 2002

### Beverage Processing

The content and quantity of phenolic compounds present in juices affects their quality, changing their color and taste and thus decreasing their value. Darkening of these products is very common due to the presence of polyphenols. In that sense, it has been demonstrated the high efficacy of laccase to remove phenols, avoiding the use of other chemical treatments such as activated carbon adsorption (Ribeiro et al., 2010). Color stability can be greatly increased after the treatment with the enzyme (Brijwani et al., 2010).

### Wine Stabilization

One of the best known applications of laccase in the food industry is in the wine stabilization through the control of phenolic compounds (Osma et al., 2010). The maintenance of flavor in this type of products is fundamental for quality control. Besides, the oxidative reactions that may occur due to the complexity of the mixture of chemicals in the wines can even intensify the color, especially in red wines (Minussi et al., 2007). Chemical methods for the elimination of phenolic compounds, such as the addition of polyvinylpolypyrrolidone (PVPP) and sulfur dioxide, have been used to stabilize the wines and prevent the loss of flavor and color quality (Minussi et al., 2002). Due laccase has good stability at low pH and it is possible to reverse its inhibition with sulfite, the treatment of wines with the enzyme is widely used (Osma et al., 2010). It has been reported that the treatment of white wine with laccase is also feasible, this represents an alternative to ensure the quality of the wines that remain stored for a long time, avoiding their deterioration and reducing the costs of their production. In addition to the advantage of using an ecological treatment (Minussi et al., 2007).

### Beer Stabilization

Another product that needs to be stored for long time periods is beer. The control of temperature, oxygen and a cloudy appearance are factors that can affect its quality (Osma et al., 2010). The presence of phenolic compounds such as proanthocyanidins can provoke the precipitation of proteins favoring haze formation (Brijwani et al., 2010). The half-life of the beer can be prolonged through laccase treatment, which can be added at the end of the production process allowing the oxidation of the phenolic compounds (Osma et al., 2010).

### Baking

Enzymes have been widely used in the bakery industry because they improve the texture of bread. Laccase is no exception and its usage has been reported to improve dough consistency and enhancing strength of gluten structures. It has also been observed how the structure of the crumb changes, the volume and softness of the dough is improved when the laccase is added (Brijwani et al., 2010).

### Sugar Beet Pectin Gelation

This product is a gel formed by the oxidative cross-linking of ferulic acid, widely used in the food industry due to its functional properties. The oxidative process can be carried out by oxidoreductases such as laccase and peroxidase. There is a fundamental difference between these enzymes, laccase only needs molecular oxygen for the oxidation process while peroxidase requires the presence of H_2_O_2_. The obtained gel after the process with laccase is thermo-irreversible and can be used in products like luncheon meat (Norsker et al., 2000; Minussi et al., 2002).

The main applications of laccase directly in food processing were aforementioned. However, alternative indirect applications have been reported. These applications may include: laccase amperometric biosensors to measure polyphenols concentration (Osma et al., 2010); the detection and quantification of ascorbic acid in different products and other biosensors for glucose and aromatic amines determination (Minussi et al., 2002). Additionally, laccase has also been applied in the synthesis of medications such as analgesic, sedatives, anti-inflammatory and antibiotics (Upadhyay et al., 2016).

## Potential Trends and Current Challenges

The use of by-products from food industry is a growing trend due to the prevailing need to combat environmental pollution and follow the trend of “reduce-reuse-recycle.” So, researchers have a shared responsibility with the private initiative to explore ways for the proper disposal of waste and its reuse in the generation of new products.

Edible mushrooms production represents one of the most important food industries around the world. As already mentioned, the production of laccase during the cultivation of different species of fungi has been proven through different studies. However, not many studies are known about the increase in laccase concentration during the industrial production of these fungi without altering their nutritional value. Which represents an area of opportunity in the optimization of the production of the fungus, while optimizing the production of laccase and even other ligninolytic enzymes. In turn, it is necessary to optimize the recovery process of the enzyme from the residual compost, explore other primary recovery techniques and purification stages that allow high purities to be achieved. This represents a real challenge, since the crude extract is very complex and not well characterized. In that sense, the integration of ultrasonication in the primary recovery of laccase using ATPS can be a suitable option for to increase the yield. It has been reported that its use can increase the yield, especially of phenolic compounds, in addition to increasing the activity; so, it could be an excellent strategy to treat the crude extract from the residual compost and evaluate its impact in the laccase activity.

Laccase production at the laboratory level in submerged cultivation has been widely reported in recent years. However, in the market there are few presentations of the enzyme, with low levels of purity and from extracts that are not fully characterized, which complicates the reproducibility of many processes that were mentioned throughout this work. Therefore, it is necessary to increase the interest in the area of production and purification of commercial laccases for the development of new products.

The potential of laccase in the pretreatment of lignocellulosic residues to integrate them into biorefinery processes in order to obtain biofuels has been well established in the literature. However, it is necessary to further consider the optimization of conditions to obtain the highest degradation yields, since in many occasions the effectiveness of the fungus is greater than the enzyme. This may be due to the presence of other ligninolytic enzymes, so the possibility of using enzyme cocktails, increase the affinity of laccase through different chemical modifications or realize changes in the parameters of the reactions that are being carried out should be explored.

Additionally, the modification of the laccase through the covalent attachment of one molecule of PEG, known as PEGylation, can be considered to improve its stability. PEGylation is common process reported for the modification of protein drugs and its efficiency has been widely demonstrated. Even though, there are few reports about laccase PEGylation, they present evidence on the improvement of its catalytic activity (Mayolo-Deloisa et al., 2015; Su et al., 2018). This can be an alternative when the process justifies it, especially when the enzyme is used for the detection of certain compounds using biosensors. In that sense, one of the applications of laccase is the detection of various compounds in certain foods. However, it is also used for the synthesis of chemical compounds, their detection and recently it has been reported that the enzyme is capable of removing morphine from aqueous systems (Huber et al., 2018). This reflects the great potential of the enzyme not only in the food industry but also for the recovery of contaminated areas, hence the importance and relevance of the information presented here.

## Concluding Remarks

Laccase is one of the most studied enzymes in the world. Its low substrate specificity and capacity to degrade phenolic compounds using just oxygen, make it suitable for different industrial applications. In addition, it is secreted by several microorganisms to degrade lignin, especially the white-rot fungi. The enzyme can be recovered from residual compost of edible mushroom as *Agaricus bisporus* and *Pleurotus ostreat*us using aqueous two-phase systems; thereby this technique can be applied for the extraction of the enzyme from other food by-products. Laccase is also capable of degrading phenolic compounds present in wastewaters from food processing such as olive oil, fermentation of sugarcane molasses, and brewing. However, laccase can also be produced using many of the agro-residues from the food industry, as lignocellulosic waste can serve as a substrate for many enzyme-producing fungi, generating a cycle of production and reuse suitable to be applied in the circular economy. This makes the enzyme highly valued in biorefinery processes for its already recognized ability to degrade lignin naturally. In addition, laccase is widely used in various food industry processes such as beverage processing, baking, stabilization of wine and beer, and sugar beet pectin gelation. This potential justifies the need to deepen the sources of production and purification processes of the enzyme. Furthermore, the opportunity to establish a complete process where the enzyme is obtained from a waste material, recovered purified and placed on the market for a new application, is still available.

## Author Contributions

KM-D and MG-G defined the general objective and the topics to be discussed. KM-D wrote and discussed the sections “Laccase as By-Product From Food Industry,” “Production of Laccase Using Agroresidues,” and “Concluding Remarks.” MG-G wrote the sections “Introduction” and “Bioprocessing of Food Industry By-Products Using Laccase.” MR-P wrote the sections “Food Industry Application of Laccase” and “Potential Trends and Current Challenges.” All authors contributed to the final review of the manuscript.

## Conflict of Interest

The authors declare that the research was conducted in the absence of any commercial or financial relationships that could be construed as a potential conflict of interest.
